# Guttiferone BL from the Fruits of *Allanblackia gabonensis* Induces Mitochondrial-Dependent Apoptosis in PA-1 Ovarian Cancer Cells

**DOI:** 10.1155/2023/8981430

**Published:** 2023-02-21

**Authors:** Aimé Gabriel Fankam, Susmita Mondal, Faustine L. M. Dongmo, Blaise Kemajou Nganou, Ingrid Simo Konga, Chitra Mandal, Victor Kuete

**Affiliations:** ^1^Department of Biochemistry, University of Dschang, P.O. Box 67, Dschang, Cameroon; ^2^Cancer Biology and Inflammatory Disorder Division, Council of Scientific and Industrial- (CSIR-) Indian Institute of Chemical Biology, 4, Raja S.C. Mullick Road, Jadavpur, 700032, Kolkata, India; ^3^Department of Zoology, Diamond Harbour Women's University, Sarisha, West Bengal 743368, India; ^4^University Institute of Technology, University of Ngaoundéré, P.O. Box 455, Ngaoundéré, Cameroon; ^5^Department of Chemistry, University of Dschang, P.O. Box 67, Dschang, Cameroon

## Abstract

Despite the recent advancement of treatment strategies, cancer ranks 2^nd^ among the causes of death globally. Phytochemicals have gained popularity as an alternate therapeutic strategy due to their nontoxic nature. Here, we have investigated the anticancer properties of guttiferone BL (GBL) along with four known compounds previously isolated from *Allanblackia gabonensis*. The cytotoxicity was assessed by 3-(4,5-dimethylthiazol-2-yl)-2,5-diphenyl tetrazolium bromide (MTT) assay. The study was extended for the assessment of the effect of GBL in PA-1 cells apoptosis induction, cell cycle distribution, and change in mitochondrial membrane potential using flow cytometry, Western blot analysis, and real-time PCR. Among the five tested compounds, GBL displayed significant antiproliferative effects against all tested human cancer cells (IC_50_ < 10*μ*M). Moreover, GBL exhibited no significant cytotoxicity towards normal ovarian epithelial cell line (IOSE 364) up to 50 *μ*M. GBL induced sub-G_0_ cell cycle arrest and significant upregulation of cell cycle regulatory proteins of ovarian cancer cell PA-1. Furthermore, GBL induced its apoptosis as depicted by the accumulation of cells both at the early and late apoptotic phase in Annexin V/PI assay. In addition, it decreased the PA-1 mitochondrial membrane potential and promoted upregulation of caspase-3, caspase-9, and Bax and downregulation of Bcl-2. GBL also showed a dose-dependent inhibition of PA-1 migration. Altogether, this study reveals that guttiferone BL, studied herein for the first time, exhibits efficient antiproliferative activity by the induction of apoptosis through the mitochondrial-dependent pathway. Its investigation as a therapeutic agent against human cancers especially ovarian cancer should be envisaged.

## 1. Introduction

The incidence of cancer is increasing, and it remains an aggressive killer worldwide despite considerable efforts. The burden of cancer rose to 19.3 million new cases and almost 10.0 million deaths in 2020 [1]. Among the three common gynecologic cancers, ovarian cancer ranks top in mortality [2]. Most of the ovarian cancer cases (up to 70%) are high-grade carcinomas that grow aggressively, metastasize rapidly, and have high chromosomal instability [3]. Though ovarian cancer has a lower occurrence rate in comparison with breast cancer, it is three times more lethal [4], and it is predicted that, by the year 2040, the mortality rate of this cancer will rise significantly [1].

To date, chemotherapy is a mainstay of cancer treatment in addition to surgery, radiotherapy, and antibody-based immunotherapy. However, the use of conventional chemicals fails due to various factors including side effects, toxicities [5], and drug resistance. In the last 10 years, novel synthetic chemotherapeutic agents have not entirely succeeded in fulfilling expectations. Therefore, there is an urgent need to develop new, effective, and affordable anticancer drugs [6].

In the last decade, herbal medicine as an important branch of complementary and alternative medicine has increasingly grown as alternative medicine for cancer treatment [7–9]. Moreover, many studies have demonstrated that natural products from plants including African flora can effectively regulate proliferation, differentiation, and expression of cancer cells [10–14]. *Allanblackia gabonensis*, a plant belonging to the family of Clusiaceae, is widely distributed in the Democratic Republic of Congo and Cameroon [15]. It is generally used as a medicinal plant to improve virility in men and to treat infections such as dysentery, colds, and toothaches [16]. The phytochemistry of *A. gabonensis* showed that it contains compounds of different classes such as xanthones, benzophenones, flavonoids, and phytosterol [17]. Past reports showed the antimicrobial and antileishmanial [17], analgesic and anti-inflammatory [18], antibacterial [19, 20], and anticancer [21] properties of its extracts and derived products.

In our continuous search of natural products to fight against cancers, this study was undertaking to investigate the cytotoxicity of polyprenylated benzophenone, guttiferone BL along with four known compounds including three flavonoids, morelloflavone, kaempferol, morelloflavone 7^″^-*O*-*β*-_D_-glucopyranoside, and one sterol, *β*-sitosterol 3-*O*-*β*-_D_-glucopyranoside, isolated in our previous study from *A. gabonensis*. The mode of action of guttiferone BL, the most active compound, was equally evaluated. To our best knowledge, this is the first time report on the anticancer potential of guttiferone BL.

## 2. Material and Methods

### 2.1. Chemicals

**Figure 1:**
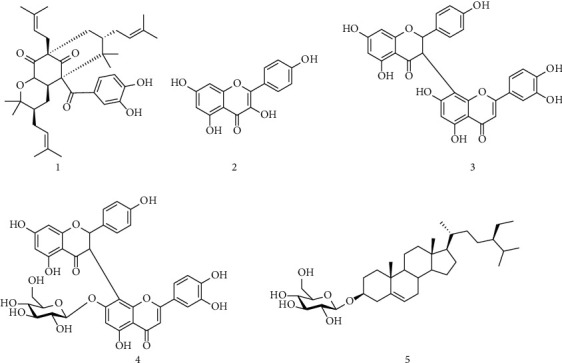
Structures of isolated compounds from *Allanblackia gabonensis.* 1: guttiferone BL; 2: kaempferol; 3: morelloflavone; 4: morelloflavone 7^″^-*O-β*-_D_-glucopyranoside; 5: *β*-sitosterol 3-*O-β*-_D_-glucopyranoside.

Mitoscreen kit (5,5′,6,6′-tetrachloro-1,1′,3,3′-tetraethylbenzimidazolylcarbocyanine iodide, JC-1), FITC-Annexin V, and CycleTEST plus DNA kit were from BD Bioscience (San Diego, USA). IMDM and MEM cell culture medium (Gibco), FBS (Gibco), antibiotic-antimycotic mixture, and trypsin–EDTA were from Invitrogen (USA). 3-(4,5-Dimethylthiazol-2-yl)-2,5-diphenyl tetrazolium bromide (MTT), propidium iodide (PI), Tween-20, and dimethyl sulphoxide (DMSO) were from Sigma-Aldrich, USA. The compounds used in the present investigation included guttiferone BL (**1 or GBL**), kaempferol (**2**), morelloflavone (**3**), morelloflavone 7^″^-*O*-*β*-_D_-glucopyranoside (**4**), and *β*-sitosterol 3-*O*-*β*-_D_-glucopyranoside (**5**) (Figure 1). The compounds were isolated from the fruits of *A. gabonensis* methanol (MeOH) extract and characterized using spectroscopic techniques coupled with a comparison of their thin-layer chromatography (TLC) profile as described by Nganou et al. [20].

### 2.2. Cell Lines and Culture Condition

Cervical cancer cells (HeLa), human glioblastoma cancer cells (U87MG), ovarian cancer cell (PA-1), and normal ovarian epithelial cell line (IOSE 364) were obtained from American Type Culture Collection (Manassas, VA). HeLa and U87MG cells were grown in IMDM, whereas PA-1 cells were grown in MEM and IOSE 364 cell line was cultured in medium MCDB 105 and M-199 in the ratio 1 : 1. All media were supplemented with 10% heat-inactivated fetal bovine serum (FBS) and 1% antibiotic-antimycotic mixture. Cells were kept at 37°C in a humidified incubator containing 5% CO_2_ and passaged twice weekly. All experiments were performed with cells at about 90% confluence.

### 2.3. Cell Viability Analysis

The cytotoxicity of the compounds was assessed by the MTT assay [22]. HeLa, U87MG, PA-1 cells (7 × 10^3^ cells/well), and IOSE 364 (1 × 10^4^) were seeded separately into 96-well plates separately. After attachment, cells were treated with compounds at various doses and incubated at 37°C in a 5% CO_2_ humidified environment for 48 h. Cell morphology was checked, and images were taken by using phase-contrast inverted microscopy (EVOS, Life Technologies). Thereafter, the medium in each well was removed and replaced by of fresh medium containing MTT (1 mg/mL). Next, the plates were incubated at 37°C for 2 h. After incubation, the supernatant was removed, and the formazan complex was dissolved with pure DMSO. The optical density was measured by ELISA reader (Thermo Fisher Scientific) at 550 nm. Cell viability was calculated from the percentage of MTT conversion in treated cells relative to untreated control cells, and cell growth inhibition for the most active compound was expressed in terms of IC_50_ values, defined as the concentration that causes 50% of inhibition of cell viability.

### 2.4. Cell Cycle Analysis

PA-1 (5 x 10^5^ cells/well) was exposed to varying concentrations of GBL for 24 h. Next, they were harvested and processed by CycleTest plus DNA kit (BD Bioscience, San Diego, USA) according to the manufacturer's instructions. Briefly, after treatment, cells were washed and incubated with trypsin solution for 10 min at 25°C followed by RNAse solution for 10 min at 25°C. Next, cells were incubated with PI solution for 10 min at 4°C in the dark. Thereafter, at least 10,000 cells were acquired using a flow cytometer (BD LSR Fortessa) and analyzed with FACSDiva 8.0.2 software.

### 2.5. Western Blotting

PA-1-untreated and PA-1-treated cells were sonicated (Qsonica-LLC, XL-2000) in ice-cold phosphate-buffered saline (PBS), and proteins were estimated using the BCA Assay Kit (Thermo Fisher Scientific). Equal amounts of proteins (80 *μ*g) were separated by sodium dodecyl sulfate-polyacrylamide gel electrophoresis (SDS-PAGE, 5–12%) and electrotransferred to nitrocellulose (NC) membrane. The NC-membrane was then blocked with Tris-buffered saline- (TBS-) bovine serum albumin (BSA; 2–5%) for 5–30 min and probed with the primary antibody (1 : 1000 dilution; Cell cycle technologies). Blots were washed with TBS–Tween-20, incubated with HRP-conjugated secondary antibodies (1 : 1000 dilution; Cell cycle technologies), and detected by Westpico ECL system. Images were taken by Bio-Rad ChemiDoc MP and evaluated with Image Lab software version 5.2.1.

### 2.6. Assessment of Changes in the Mitochondrial Membrane Potential

Alterations in the mitochondrial membrane potential (*ΔΨ*m) were determined quantitatively by flow cytometry, using JC-1 dye (Invitrogen, USA) at 24 h posttreatment as previously described [21]. JC-1 accumulates within the intact mitochondria to form J-aggregates that resulted in a change of fluorescence from red to green indicating decreased *ΔΨ*m. Briefly, PA-1 cells (5 × 10^5^ cells/well) in a 6-well plate were exposed to GBL for 24 h, then washed in PBS and incubated with JC-1 (25 *μ*M) for 30 min in the dark at 37°C. The percent positive cells with green fluorescence (JC-1 monomers) which represented polarized cells were measured [23]. The experiment was performed using a flow cytometer (BD LSR Fortessa) and analyses with FACS Diva 8.0.2 software. At least 10,000 cells were analyzed for this experiment.

### 2.7. Annexin V and PI Assay

Externalizations of phosphatidylserine were verified by double staining the cells with Annexin V-FITC and PI as previously described [24]. Cells (5 × 10^5^ cells/well) were treated with different concentrations of GBL. After 24 h incubation at 37°C in 5% CO_2_, cells were washed with phosphate-buffered saline (PBS), resuspended in the Annexin V binding buffer according to the manufacturer's instructions, and incubated for 45 min at 25°C. Cells were further incubated with Annexin V-FITC and PI for 20 min at 4°C in the dark. Data acquisition was done using a flow cytometer (BD LSR Fortessa) and analyzed with FACSDiva 8.0.2 software. At least 10,000 cells were analyzed for this experiment.

### 2.8. RT-PCR Analysis of Apoptotic Genes

Total cellular RNA was extracted from GBL-treated and GBL-untreated cells (1 × 10^6^ cells/well) using an RNeasy Mini Kit (Qiagen), and 1 *μ*g of extracted RNA were reverse transcribed into complementary DNA (cDNA) with random primers using transcription system (Promega) according to the manufacturer's protocol. Polymerase Chain Reaction (PCR) of caspase-3, caspase-9, Bcl2, and Bax genes were carried out with specific forward and reverse primers using a PTC-100 system (MJ Research). The details of primers are listed in Table S1 (Supplementary material). Glyceraldehyde-3-phosphate dehydrogenase (GAPDH) was used as an internal control. The PCR products obtained were electrophoresed on an agarose gel (1%), which was stained with ethidium bromide (EtBr) and visualized under UV light. The signal intensity of the respective DNA bands was measured with ImageJ software v 1.50i.

### 2.9. Scratch Wound Assay

Scratch wound assay was carried out as previously described [25] with slight modifications. PA-1 cells were plated in 6-well plates with >90% confluence. Scratch wounds were made with a micropipette tip, washed thrice to remove the floating cells, and treated with GBL at IC50 dose in FBS free medium and incubating them. Images were taken at 0 hrs, 8 hrs, and 24 hrs. The width of the wounds was estimated from five different fields of three separate experiments for untreated and treated groups, and percentage wound healing was calculated from the width at 8 and 24 hrs versus the initial width at 0 hrs, all using ImageJ software.

### 2.10. Statistical Analysis

Most of the data shown in this study were representative of three sets of independent experiments. The results were represented as the mean ± standard deviation from independent experiments.

## 3. Results

### 3.1. Screening of Antiproliferative Activity of an Array of Compounds

**Figure 2:**
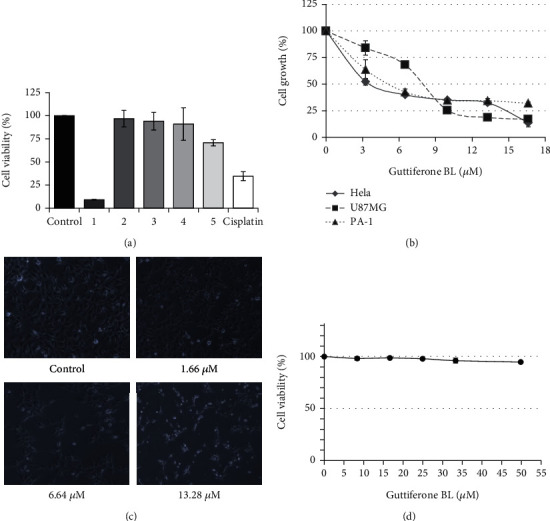
Antiproliferative activity of guttiferone BL. (a) Viability of Hela cells in the presence of the different compounds isolated from *A. gabonensis*; 1: guttiferone BL (17 *μ*M); 2: kampherol (35 *μ*M); 3: morelloflavone (18 *μ*M); 4: morelloflavone 7^″^-*O*-*β*-_D_-glucopyranoside (19 *μ*M); 5: *β*-sitosterol 3-*O*-*β*-_D_-glucopyranoside (14 *μ*M). Cisplatin (5 *μ*M) was used as control drug. (b) Guttiferone BL induces a dose-dependent inhibition of HeLa, PA-1, and U87-MG cancer cell lines after 48 h incubation. (c) Morphological examination of PA-1 cells after treatment with various concentrations of GBL for 48 h showed inhibition of cellular proliferation (magnification ×20). (d) Guttiferone BL exhibited no cytotoxicity to normal ovarian epithelial cell (IOSE 364) after 48 h incubation.

Previously, our lab has evaluated the antiproliferative activity of an array of extracted samples preliminary on cervical cancer cell line HeLa. Among them, the methanol extract from the fruits of *A. gabonensis* as well as their ethyl acetate and hexane fractions showed the maximum potency of anticancer activity in HeLa cell line [21]. Here, we have studied five compounds (Figure 1) isolated from the fruit's methanol extract of *A. gabonensis* for their antiproliferative activity on HeLa. Among them, guttiferone BL (GBL) showed the maximum efficacy against HeLa at 17 *μ*M for 48 h (more than 80% growth inhibition) (Figure 2(a)).

### 3.2. Guttiferone BL Is a Potent Antiproliferative Compound

Based on these results, guttiferone BL has been selected and tested on three different cancer cell lines, namely, Hela, U87-MG, and PA-1 for the determination of its IC_50_ values. Dose-dependent inhibition of cell proliferation was observed in all these cancer cell lines after 48 h of GBL treatment (Figure 2(b)). The IC_50_ values obtained were 3.99 *μ*M, 5.00 *μ*M, and 7.99 *μ*M towards HeLa, PA-1, and U87-MG cancer cell lines, respectively. The results are summarized in Table 1. Significant inhibition of PA-1 cell growth as well as attainment of rounded shape and cluster formation upon treatment with GBL at 6.64 *μ*M and 13.28 *μ*M was observed (Figure 2(c)). However, GBL showed no significant cytotoxicity towards normal ovarian epithelial cell line (IOSE 364) up to 10 × IC_50_ value of ovarian cancer cell line (Figure 2(d)).

### 3.3. Guttiferone BL Induced Sub-G_0_ Cell Cycle Arrest in PA-1 Cells

**Figure 3:**
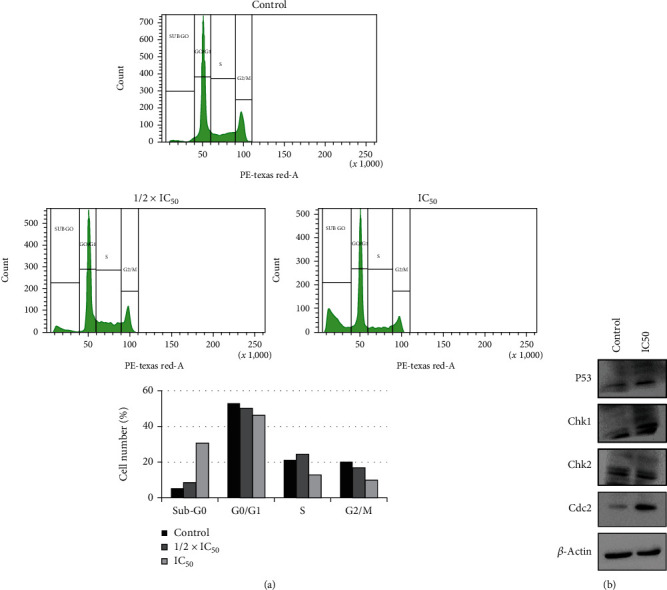
Guttiferone BL induces apoptosis in PA-1 cells. (a) Histogram plots showing GBL induces accumulation of PA-1 cells in sub-G_0_ phase after treatment and incubation for 24 h; graphical representation of cell cycle analysis showing percentage of cells arrested in sub-G_0_ phase. (b) GBL treated PA-1 cells exhibited upregulation of P53, Chk1, Chk2, and cdc2 indicating cell cycle arrest. IC_50_ = 5.00 ± 0.70 *μ*M.

Considering the results obtained, we have investigated whether cell death detected would be due to apoptosis induction. First, we checked the cell cycle status of ovarian cancer cell line upon GBL treatment and analyzed by a flow cytometer. The results revealed that GBL induced a concentration-dependent accumulation of the cell population in sub-G_0_ after 24 h treatment. Cell cycle analysis exhibits a significant arrest of 8.5% cells in sub-G_0_ phase at 1/2 × IC_50_ dose and 30.7% at IC_50_ dose, against 5.4% for untreated cells. A concomitant decrease of G0/G1, S, and G2/M populations in PA-1 cells was also observed (Figure 3(a)). Moreover, we assessed a few cell cycle regulatory proteins through Western blotting analysis. GBL-treated PA-1 cells exhibited upregulation of P53, Chk1, Chk2, and cdc2 (Figure 3(b)). *β*-Actin served as a loading control.

### 3.4. Guttiferone BL Induced Apoptosis in Ovarian Cancer through the Intrinsic Pathway

**Figure 4:**
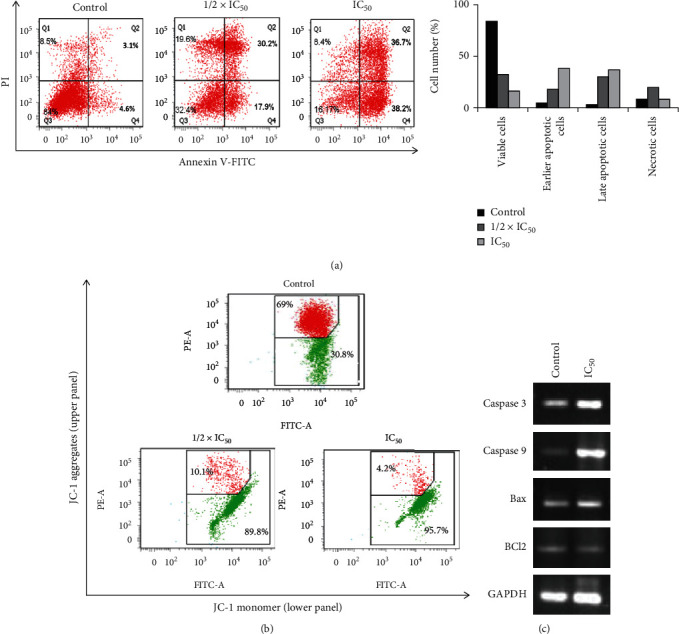
GBL induces intrinsic apoptotic pathway in PA-1 cells. (a) Annexin V/PI apoptotic assay showed that GBL induces early and late apoptosis in PA-1 cells in a dose-dependent manner. (b) GBL induces mitochondrial membrane depolarization in PA-1 cells (polarized cells: JC-1 aggregated red fluorescence; depolarized cells: JC-1 monomer green fluorescence). (c) Representative agarose gel blots showed upregulation of apoptosis-associated genes in GBL-treated PA-1 cells. IC_50_ = 5.00 ± 0.70 *μ*M.

Sub-G_0_ cell cycle arrest is an indication of cell death. Therefore, we wanted to check whether GBL induces apoptosis in ovarian cancer. To do so, we performed Annexin V/PI apoptotic assay using flow cytometry. Annexin V/PI-positive cells were recorded by a flow cytometer (including the early and late apoptosis, Q_2_ and Q_4_) were 7.7% (control), 48.1% (1/2 × IC_50_), and 74.19% (IC_50_) (Figure 4(a)). These results indicated that guttiferone BL induced apoptosis in a concentration-dependent manner.

Changes in the mitochondrial membrane potential (*ΔΨ*m) are observed during the intrinsic pathway of apoptosis. Therefore, we wanted to check the effect of GBL on mitochondrial depolarization. Data obtained in this study showed that GBL induced a concentration-dependent mitochondrial membrane depolarization in PA-1 cells (Figure 4(b)). These results suggest the association of the mitochondrial pathway in GBL apoptotic cell death in PA-1 cells.

Further, we assessed the expression of a few selected apoptotic-associated genes (caspase-3 and -9, Bax and Bcl-2) by RT-PCR. The results demonstrated that GBL promoted a remarkable up-regulation of caspase-3, caspase-9, (panel A), and Bax (panel B) gene expressions and a down-regulation of Bcl-2 (panel B) gene compared to untreated cells. GAPDH served as a loading control (Figure 4(c)). This indicates that GBL induces apoptosis in the intrinsic-dependent pathway in PA-1 cells.

### 3.5. Guttiferone BL Inhibited Cellular Migration in PA-1 Cells

**Figure 5:**
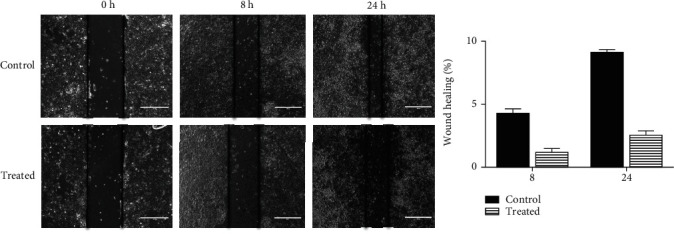
Inhibition of the cellular migration of PA-1 cancer cells. PA-1 cells were plated in 6-well plates with >90% confluence. Scratch wounds were made with a micropipette tip, washed thrice, and treated with IC_50_ dose (5.00 ± 0.70 *μ*M) of GBL in medium free FBS and incubated for 0, 8, and 24 hours. The wound width was measured for untreated and treated groups from at least five different fields of three separate experiments, and percentage wound healing was calculated from the width at 8 and 24 hrs versus the initial width at 0 h, all using ImageJ software.

Further, the effect of the guttiferone BL on cell migration was tested by scratch wound assay. PA-1 cells were plated in 6-well plates with >90% confluence. Scratch wounds were made with a micropipette tip, washed thrice to remove the floating cells treated with IC_50_ dose of guttiferone BL in medium, and incubated for 0, 8, and 24 hours. The results showed that guttiferone BL inhibited the cellular migration of PA-1 cancer cells (Figure 5).

## 4. Discussion

Despite considerable efforts, cancer remains an aggressive killer worldwide. In the last decade, natural products and mainly those from plant sources have received increasing attention for their potential as a novel cancer preventive and therapeutic agents [7–9, 26–30]. A threshold of IC_50_ ≤ 10 *μ*M after 48 h incubation has been set to identify compounds having significant or strong cytotoxicity [27, 31]. Following our previous study which showed that methanol extract, as well as their ethyl acetate and hexane fractions from fruits of *A. gabonensis*, have significant activity against HeLa cancer cells [21], we have examined five compounds from the fruit's methanol extract of *A. gabonensis* for their antiproliferative activity. Results showed that guttiferone BL exerts significant anti-proliferative activity (IC_50_ < 10 *μ*M) against the tested cancer cell lines. Moreover, GBL was nontoxic towards normal ovarian epithelial cells indicating that it exhibits some cytotoxic specificity. Some previous studies revealed that several polyprenylated benzophenones found in the Clusiaceae family have potent biological activities, especially cytotoxicity against cancer cell lines [32, 33].

Benzophenones were reported to inhibit cancer cell lines through various mechanisms of action, including apoptosis, cell cycle arrest, and endoplasmic reticulum response [32]. For instance, guttiferone E, xanthochymol, and guttiferone H isolated from *Garcinia xanthochymus* have shown to induce the cell cycle arrest, caspase activation associated with interference of the mitochondrial membrane potential, and the activation of the endoplasmic reticulum stress [34]. Furthermore, isogarcinol, isoxanthochymol, and guttiferone E were shown to strongly induce apoptosis in the leukemia cell line CCRF-CEM through activation of caspase-3/caspase-7, caspase-8, and caspase-9 [13].

Apoptosis, a major form of programmed cell death, is a defense mechanism and a tumor suppressor pathway essential for the development and maintenance of cellular homeostasis. Deregulated apoptosis leads to resistance to chemo- and radiotherapy [35]. Most chemotherapeutic agents induce cancer cell death by the activation of the apoptotic pathway. A limited number of FDA-approved anticancer agents directly target apoptotic pathways [36]. Such targeted therapy against cancer has become important. Earlier studies have reported that several phytochemicals can suppress the growth of cancer cells through disruption of cell cycle progression [37, 38]. Moreover, cell cycle arrest at sub-G_0_ phase with an increasing cell population indicates apoptosis [39, 40]. In this study, cell cycle analysis by a flow cytometer of PA-1 cells revealed that GBL induced a concentration-dependent accumulation of the cell population in sub-G_0_ after 24 h treatment (Figures 3(a) and 3(b)). These results indicate that GBL induces cell death in PA-1-treated cells. During early apoptosis phosphatidylserine are exposed on the external surface of the cell membrane, which can be assessed using the simultaneous staining of cells with FITC-Annexin-V/PI [41]. In this study, Annexin V/PI-positive cells were recorded, indicating that GBL resulted in the promotion of apoptosis. Loss of mitochondrial membrane potential (*Δψ*m) is an indicator of onset of apoptosis, which constitutes an irreversible checkpoint during apoptosis [42, 43]. These results suggest that GBL induces mitochondrial-dependent apoptosis in PA-1 cells. Caspase-3 plays an important role in the execution phase of apoptosis and its activation and subsequent cleavage of a set of important cellular proteins leading to the appearance of apoptotic morphology [44]. It is also known that caspase-9 induces loss of mitochondrion membrane concomitant with the *Bcl-2* and *Bcl-xL* cleavage [45, 46]. Upregulation of Bax is also an important parameter that characterized mitochondrial depending apoptosis [47]. The results demonstrated that GBL promoted a remarkable upregulation of caspase-3, caspase-9 gene expressions, and a downregulation of Bcl-2 gene compared to untreated cells (Figure 4(c)).

Cellular migration provides the influence of metastasis to the cancer cells. GBL exhibited a significant reduction of cellular migration (Figure 5) and thereby inhibiting the metastatic property of ovarian cancer.

## 5. Conclusions

In this study, the cytotoxic effects of guttiferone BL along with five known compounds isolated from the methanol extract of *Allanblackia gabonensis* were investigated. Guttiferone BL treatment inhibited the growth of three different cancer cell lines with various mutation and drug-resistant properties. Furthermore, it induced apoptosis in ovarian cancer cells through the mitochondrial pathway. This study demonstrates for the first time the anticancer potential of guttiferone BL; thus, its investigation as a therapeutic agent for the treatment of human cancers especially ovarian cancer should be envisaged.

## Figures and Tables

**Table 1:** Summary of the antiproliferative activity of guttiferone BL.

Cancer/normal cell type	Cell lines	^a^IC50 values (*μ*M)
Cervical	HeLa	3.69 ± 0.36
Grade IV glioblastoma	U87-MG	7.99 ± 0.03
Ovarian	PA-1	5.00 ± 0.70
Normal ovarian epithelial cell line	IOSE 364	>50

^a^IC50: inhibitory concentration 50 or IC50 represents the concentration at which a substance exerts half of its maximal inhibitory effect.

## Data Availability

All the important data generated or analyzed during this study are included in this published article.

## Supplementary Materials

Supplementary Materials Table S1: the primer details.
