# Supplementary material for: Guttiferone BL from the Fruits of Allanblackia gabonensis Induces Mitochondrial-Dependent Apoptosis in PA-1 Ovarian Cancer Cells
Source: Biomed Res Int. 2023 Feb 21;2023:8981430. doi: 10.1155/2023/8981430 (PMC9974259; doi:10.1155/2023/8981430)
Supplement: Supplementary Materials — Table S1: the primer details. [file 8981430.f1.docx]

**Guttiferone BL from the fruits of *Allanblackia gabonensis* induces mitochondrial-dependent apoptosis in PA-1 ovarian cancer cells**

*Aimé Gabriel Fankam^1,2*^, Susmita Mondal^3^, Faustine L. Dongmo Mafodong ^4^, Blaise Kemajou Nganou ^5^, Ingrid Simo Konga^5^, Chitra Mandal^2,3^, Victor Kuete^1**^*

*^1^ Department of Biochemistry, University of Dschang, P.O. Box 67, Dschang, Cameroon.*

*^2^ Cancer Biology and Inflammatory Disorder Division, Council of Scientific and Industrial (CSIR)-Indian Institute of Chemical Biology, 4, Raja S.C. Mullick Road, Jadavpur, Kolkata-700032, India.*

*^3^ Department of Zoology, Diamond Harbour Women’s University, Sarisha, West Bengal 743368, India.*

*^4^ University Institute of Technology, University of Ngaoundéré, P.O. Box 455, Ngaoundéré, Cameroon.*

*^5^ Department of Chemistry, University of Dschang, P.O. Box 67, Dschang, Cameroon.*

**Author’s contacts.**

*Susmita Mondal:* [*susmita.blysci@gmail.com*](mailto:susmita.blysci@gmail.com)

*Faustine L. Dongmo Mafodong:* [*dongmomafo@yahoo.fr*](mailto:dongmomafo@yahoo.fr)

*Blaise Kemajou Nganou:* [*nganoublaise@yahoo.fr*](mailto:nganoublaise@yahoo.fr)

*Ingrid Simo Konga:* [*simoingrid@yahoo.fr*](mailto:simoingrid@yahoo.fr)

*Chitra Mandal:* [*chitra_mandal@yahoo.com*](mailto:chitra_mandal@yahoo.com)

**Corresponding author:**

**Tel: +237 677536510 ; E-mail :* [*agfankam@yahoo.fr*](mailto:agfankam@yahoo.fr)*; P.O. Box 67 Dschang, Cameroon (Dr. Aimé Gabriel Fankam). ORCID :* [*0000-0001-7008-7453*](https://orcid.org/0000-0001-7008-7453)*.*

**Tel: +237 677355927; E-mail: [kuetevictor@yahoo.fr](mailto:kuetevictor@yahoo.fr); P.O. Box 67 Dschang, Cameroon (Prof. Dr. Victor Kuete). *ORCID :* [*0000-0002-1070-1236*](https://orcid.org/0000-0002-1070-1236)

**Table S1.** The primer details

| **^a^Gene name** | **Primer sequence** | **^b^Tm (^0^C)** | **Product size (bp)** |
| --- | --- | --- | --- |
| BAX Leftprimer | 5’-GGGGACGAACTGGACAGTAA-3’ | 54 | 490 |
| BAX Right primer | 5’-CCTCCCAGAAAAATGCCATA-3’ | 50 |  |
| BCL2 Leftprimer | 5’-GGATGCCTTTGTGGAACTGT-3’ | 52 | 457 |
| BCL2 Right primer | 5’-GGTGCTTGGCAATTAGTGGT-3’ | 52 |  |
| Caspase 3 Leftprimer | 5’-TGGAATTGATGCGTGATGTT-3’ | 48 | 403 |
| Caspase 3 Right primer | 5’-TCAAGCTTGTCGGCATACTG-3’ | 52 |  |
| Caspase 9 Left primer | 5’-GCTTAGGGTCGCTAATGCTG-3’ | 54 | 480 |
| Caspase 9 Right primer | 5’-GTGCTGAACATCCCACAATG-3’ | 52 |  |
| GAPDH Leftprimer | 5’-GGAAGGACTCATGACCACAG-3’ | 56 | 225 |
| GAPDH Right primer | 5’-GTCAGGTCCACCACTGACAC-3’ | 56 |  |

**^a^BAX :** Bcl-2-Associated X protein; **BCL2**: B-cell lymphoma 2; Caspase: Cysteine Aspartases; **GAPDH** : Glyceraldehyde 3-phosphate dehydrogenase. **^b^Tm** : melting temperature.
